# Supplementary material for: Rab11b-mediated integrin recycling promotes brain metastatic adaptation and outgrowth
Source: Nat Commun. 2020 Jun 15;11:3017. doi: 10.1038/s41467-020-16832-2 (PMC7295786; doi:10.1038/s41467-020-16832-2)
Supplement: Supplementary file 1 — Supplementary Information [file 41467_2020_16832_MOESM1_ESM.pdf]

Supplementary Information

Rab11b-mediated integrin recycling promotes brain metastatic adaptation and outgrowth

Howe *et al*

a

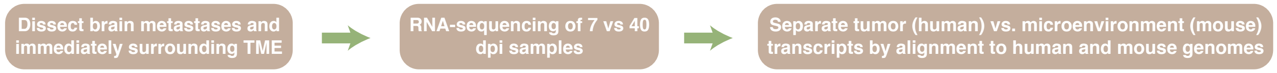

b

		Mapped Reads (number, percent)		Non-duplicated Reads (number, percent)		Uniquely Mapped Reads (number)		
	**Reads (number)**	**Human Genome**	**Mouse Genome**	**Human Genome**	**Mouse Genome**	**Human Genome**	**Mouse Genome**	**Non-unique**
7 dpi 1	281,110,000	8,143,705 (2.90)	167,787,638 (59.69)	7,188,232 (88.27)	119,411,916 (71.17)	299,492	112,523,176	6,888,740
7 dpi 2	274,135,006	7,126,491 (2.60)	170,311,610 (62.13)	6,085,154 (85.39)	124,963,057 (73.37)	302,585	119,180,488	5,782,569
7 dpi 3	281,117,650	4,003,006 (1.42)	166,052,388 (59.07)	3,418,253 (85.39)	131,210,530 (79.02)	141,143	127,933,420	3,277,110
40 dpi 1	49,147,630	4,858,961 (9.89)	24,788,916 (50.44)	4,418,328 (90.93)	21,369,108 (86.20)	4,073,866	21,024,646	344,462
40 dpi 2	35,736,586	3,197,722 (8.95)	18,411,620 (51.52)	2,948,679 (92.21)	15,473,857 (84.04)	2,519,255	15,044,433	429,424
40 dpi 3	37,048,963	1,690,759 (4.56)	20,685,577 (55.83)	1,515,874 (89.66)	17,488,554 (84.54)	1,067,413	17,040,093	448,461
40 dpi 4	29,327,433	3,116,383 (10.63)	15,137,727 (51.62)	2,847,952 (91.39)	12,495,962 (82.55)	2,532,070	12,180,080	315,882
40 dpi 5	25,120,189	708,020 (2.82)	12,660,032 (50.40)	550,577 (77.76)	12,077,288 (95.40)	438,468	11,965,179	112,109
40 dpi 6	30,049,331	1,074,674 (3.58)	13,738,601 (45.72)	861,488 (80.16)	13,576,870 (98.82)	721,846	13,437,228	139,642
40 dpi 7	20,111,560	7,668,750 (38.13)	4,614,000 (22.94)	7,159,522 (93.36)	3,618,578 (78.43)	6,906,262	3,365,318	253,260
40 dpi 8	23,434,168	8,181,842 (34.91)	6,203,042 (26.47)	7,561,314 (92.42)	5,187,666 (83.63)	7,443,485	5,069,837	117,829
40 dpi 9	21,030,675	2,237,077 (10.64)	10,800,326 (51.36)	2,050,681 (91.67)	9,191,897 (85.11)	1,797,086	8,938,302	253,595

c

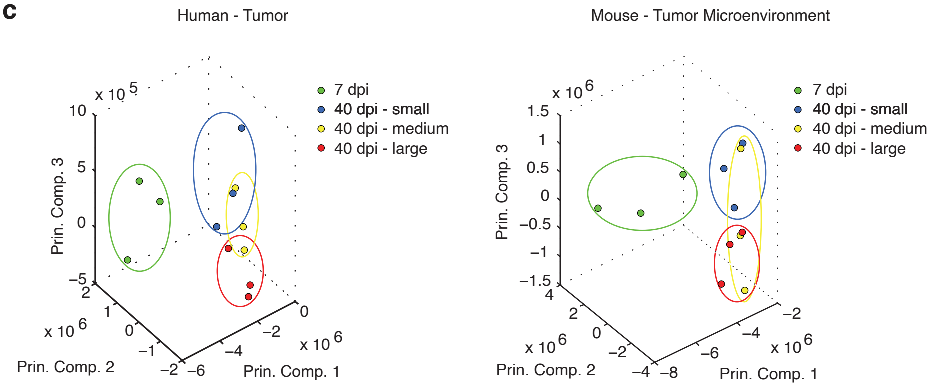

d

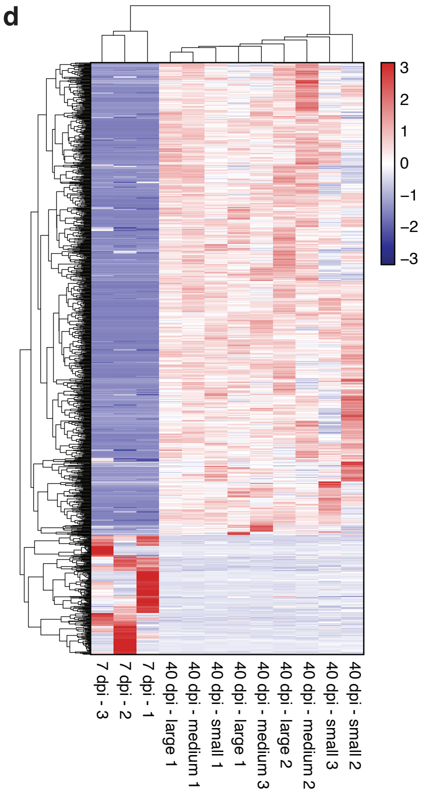

Supplementary Figure 1. RNA-sequencing of single cell versus overt breast cancer brain metastases. Related to Figure 1.
a, Schematic of experimental and bioinformatic procedure.
b, Table of reads per sample, aligned to human or mouse genome, before and after separation based on unique alignment.
c, PCA analysis of human (left) and mouse (right) RNA-sequencing samples.
d, Heatmap of 1015 genes that were significantly differentially expressed between 7 and 40 dpi samples with a q-value less than 0.05.

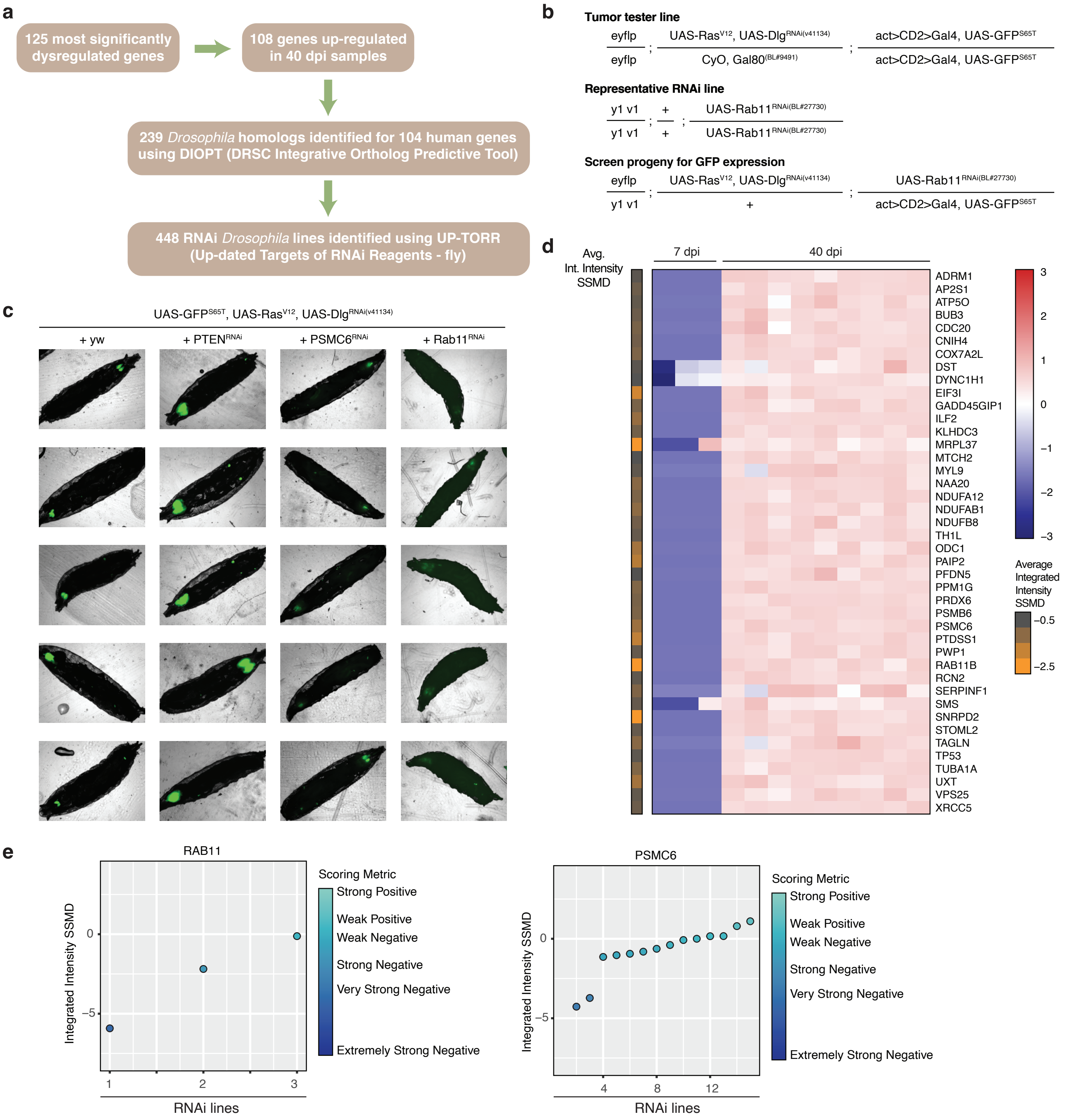

Supplementary Figure 2. *Drosophila melanogaster* model characterization and selection of hit genes. Related to Figure 1.
a, Schematic of identification of *Drosophila* orthologs and RNAi lines.
b, Genotype of the tumor tester line.
c, Representative images showing tumor signal (GFP positive, green) in larvae from negative control (yw), positive control (shPTEN), and two hit genes (PSMC6 and Rab11).
d, Heatmap showing expression of hit genes in original human RNA sequencing data, annotated with the average strictly standardized mean difference (SSMD) of the integrated intensity from the *Drosophila* screen.
e, All integrated intensity SSMD data for two representative hit genes.

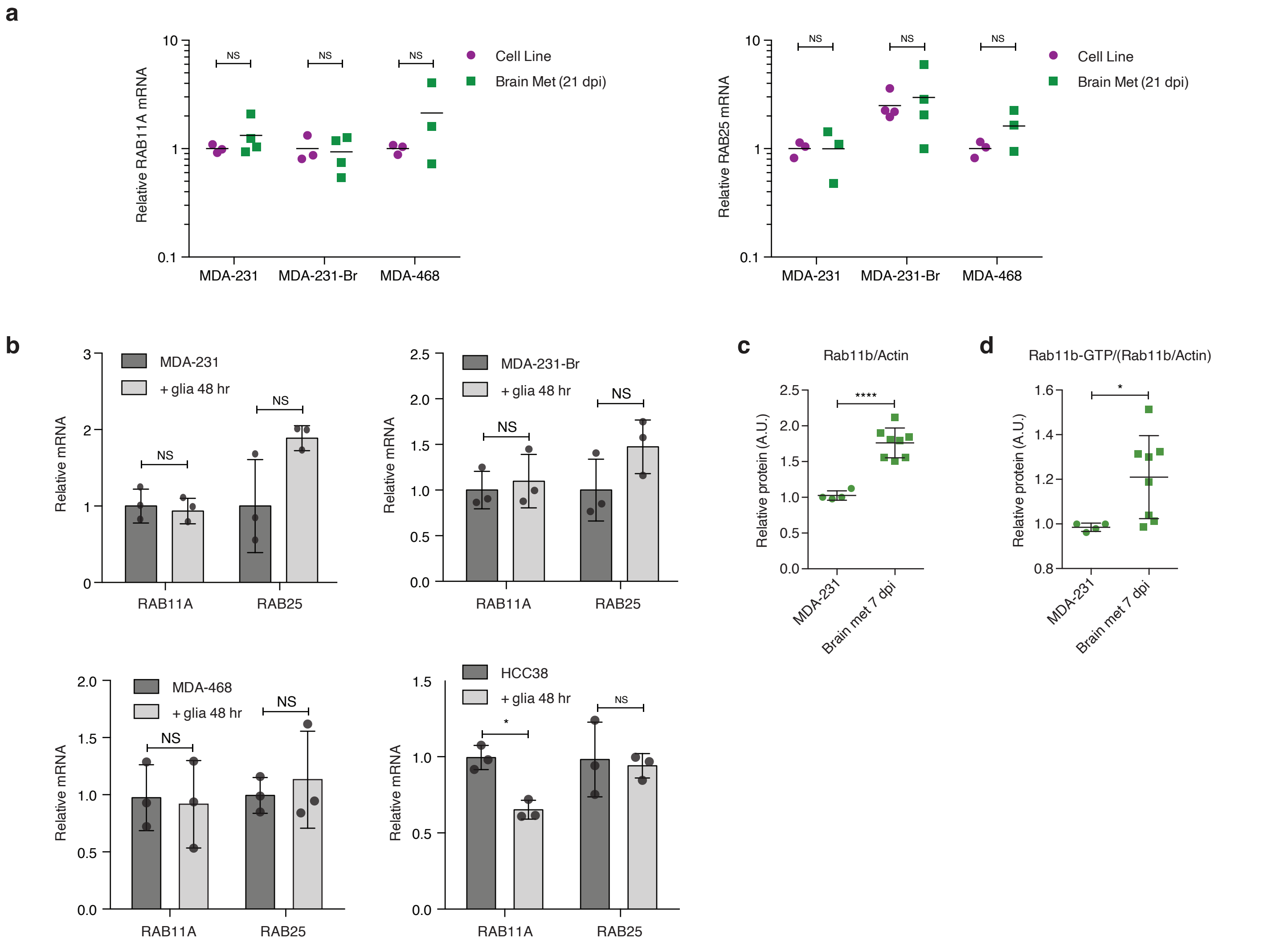

Supplementary Figure 3. Only the Rab11 B isoform is up-regulated by the brain metastatic microenvironment. Related to Figure 2.
a, qPCR for Rab11a and Rab25 in cells grown in culture versus brain metastases. All values are normalized to MDA-231 cells in culture. n = 3. Student's t-test.
b, qPCR for Rab11a and Rab25 in cell lines co-cultured with primary murine glia or CAF cells for 2 days. Mean ± s.d. All values normalized to single culture. n = 3. Student's t-test.
c-d, Quantification of two independent experiments, related to Figure 2H. *Points*, four or seven individual biological samples from two independent experiments. Mean ± s.d. Two-tailed t-test. (C) Rab11b divided by actin for each sample. Samples from each blot normalized to MDA-231. (D) Rab11b-GTP divided by Rab11b/actin ratio shown in (C). Samples from each blot normalized to MDA-231.
For all panels, * $p < 0.05$, **** $p < 0.001$.

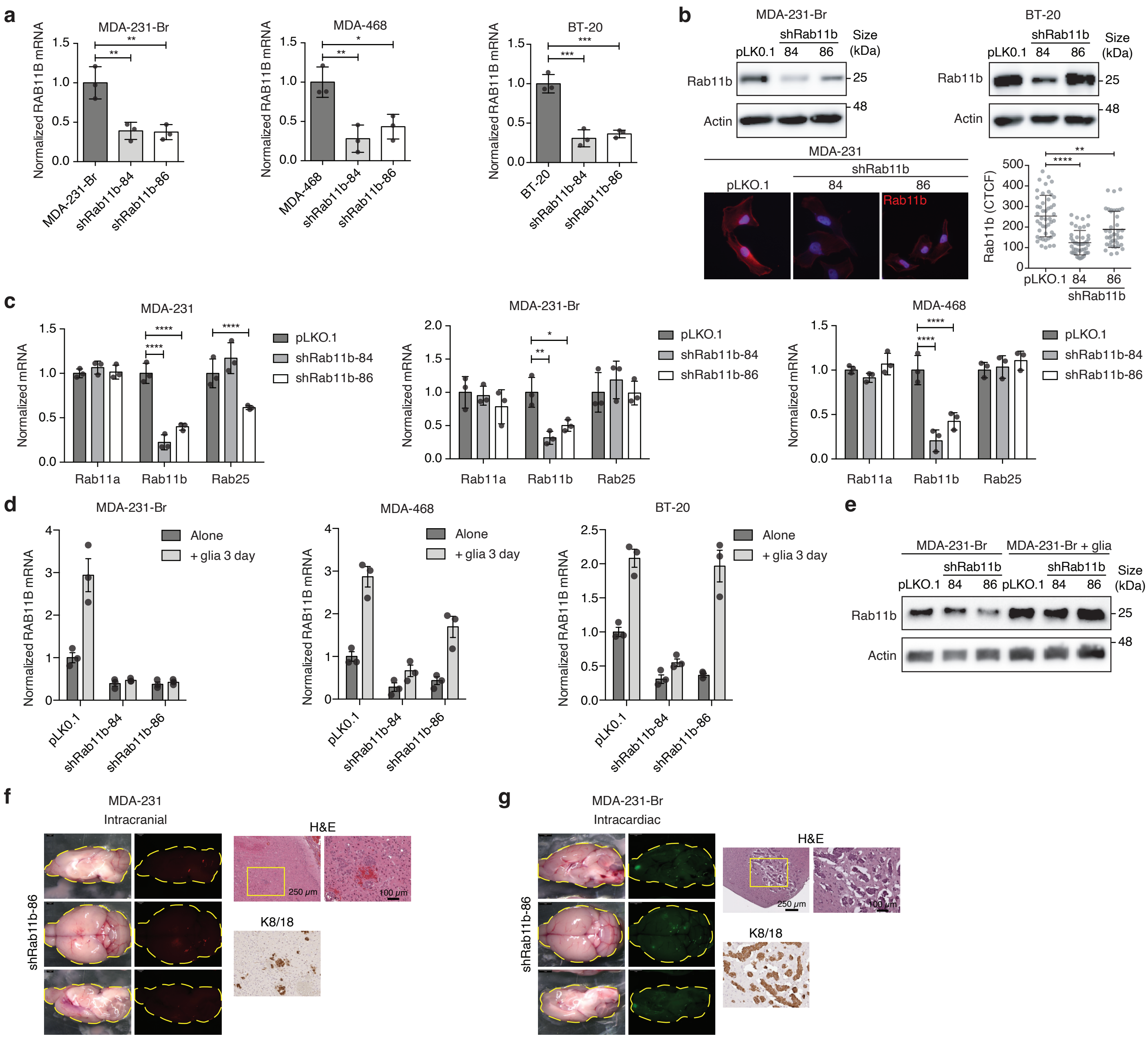

Supplementary Figure 4. Rab11b knockdown prevents glial-mediated Rab11b up-regulation.

a, Normalized mean RAB11B expression in cells expressing indicated constructs, relative to pLKO.1 empty vector. Mean ± s.d. n = 3. ANOVA with Dunnett's multiple comparison test.

b, Rab11b immunoblots (top) and immunocytochemistry (bottom) for cells expressing indicated constructs. n = 3. Mean ± s.d. Two-way ANOVA with Tukey's multiple comparison test.

c, Rab11 isoform expression in cells expressing indicated constructs. For each isoform, data presented relative to pLKO.1. Mean ± s.d. n = 3. ANOVA with Dunnett's multiple comparison test.

d, Normalized RAB11B expression in cells expressing indicated constructs cultured alone or with primary murine glia for three days, relative to pLKO.1 alone. Mean ± s.d. n = 3. Two-way ANOVA with Tukey's multiple comparison test.

e, Rab11b immunoblots for cells expressing indicated constructs cultured alone or with primary murine glia for five days followed by removal of glial cells using magnetic bead-based stromal cell depletion.

f, Representative images of mice intracranially injected with MDA-231-tdTomato shRab11b-86 cells. Mice were sacrificed 24 dpi, imaged, and H&E and immunohistological staining performed for cytokeratin 8/18 (K8/18).

g, Representative images of mice intracranially injected with MDA-231-Br-EGFP shRab11b-86 cells. Mice were sacrificed 28 dpi, imaged, and H&E and immunohistological staining performed for cytokeratin 8/18 (K8/18).

For all panels, * $p < 0.05$, ** $p < 0.01$, *** $p < 0.001$, **** $p < 0.0001$.

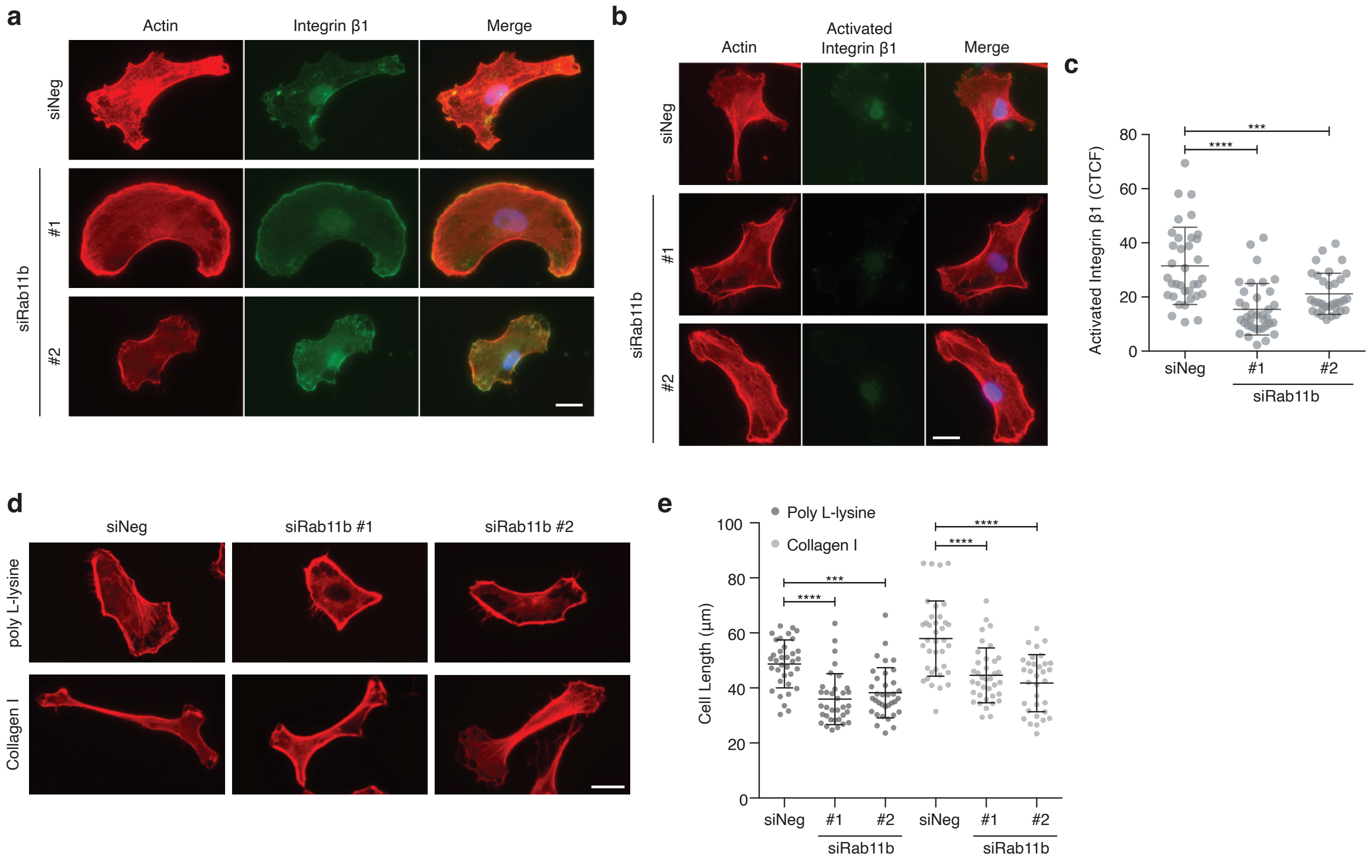

Supplementary Figure 5. siRab11b decreases active integrin β1 and cell spreading. Related to Figure 4.
a, MDA-231 cells stained for integrin β1 and actin (phalloidin, to delineate cell boundaries). Scale bar 20 μm.
b, MDA-231 cells stained for active integrin β1 and actin (phalloidin, to delineate cell boundaries). Scale bar 20 μm.
c, Corrected total cellular active integrin β1 fluorescence (CTCF) determined for individual cells. Mean ± s.d. ANOVA with Tukey's multiple comparison test.
d, MDA-231 cells plated on poly L-lysine or Collagen I and stained for actin (phalloidin, red) and nuclei (DAPI, blue). Scale bar 20 μm.
e, Quantification of cell length. Mean ± s.d. ANOVA, Tukey's multiple comparison test
For all panels, * $p < 0.05$, ** $p < 0.01$, *** $p < 0.001$, **** $p < 0.0001$.

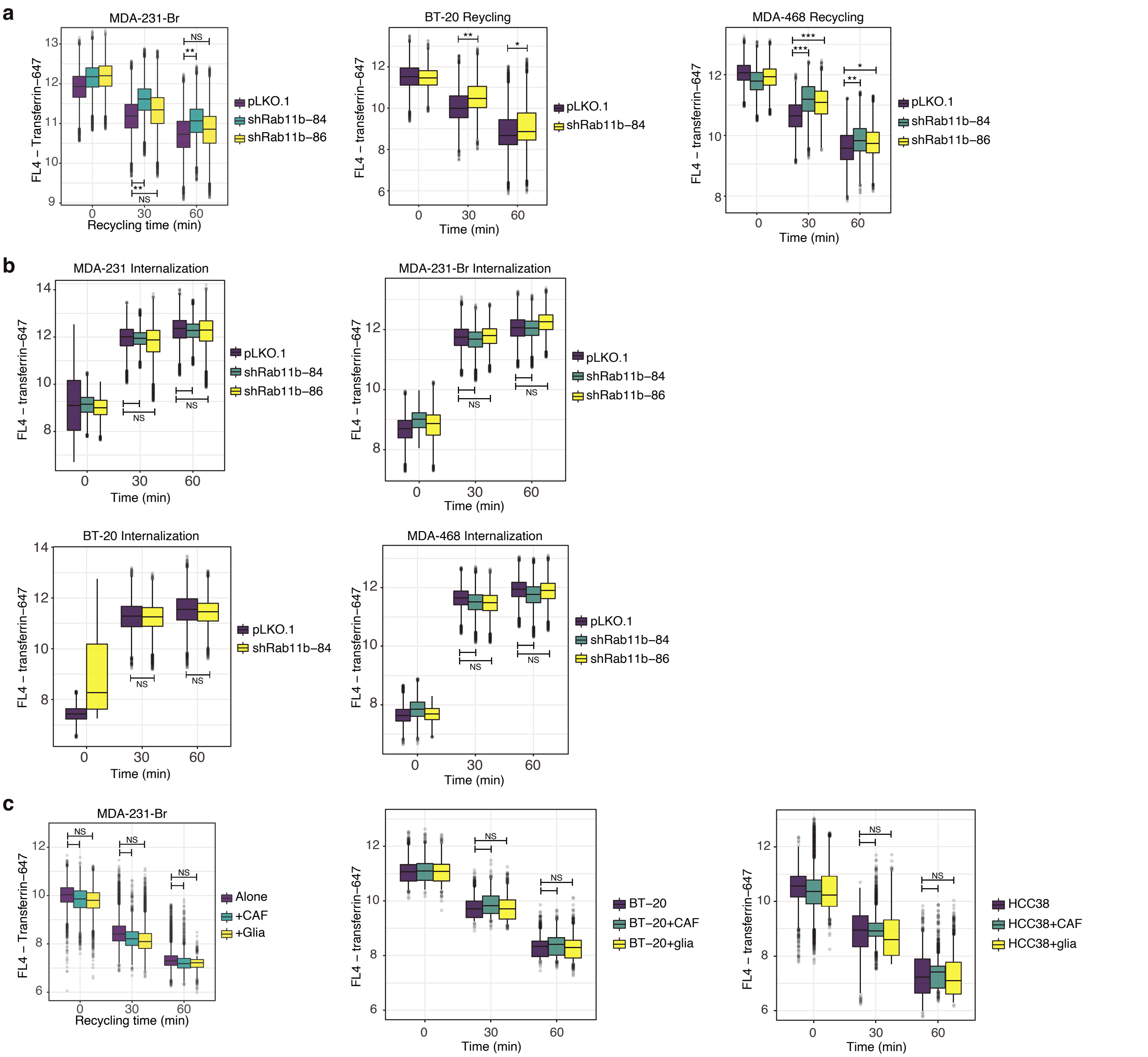

Supplementary Figure 6. Internalization and recycling of the transferrin receptor. Related to Figure 4.

a, Recycling of the transferrin receptor. BT-20, ANOVA with Tukey's multiple comparison test. MDA-468, two-way ANOVA with Sidak's multiple comparison test. n = 2 independent experiments. Boxes, first to third interquartile range, line, mean, points, outliers.

b, Internalization of the transferrin receptor. MDA-231, MDA-231-Br, MDA-468, two-way ANOVA with Sidak's multiple comparison test. BT-20, ANOVA with Tukey's multiple comparison test. n = 2 independent experiments. Boxes, first to third interquartile range, line, mean, points, outliers.

c, Cells were co-cultured for two days then recycling assay performed as in A. Cancer cells were selected on expression of CD-44. n = 2 independent experiments. Boxes, first to third interquartile range, line, mean, points, outliers. Two-way ANOVA with Sidak's multiple comparison test.

For all panels, * $p < 0.05$, ** $p < 0.01$, *** $p < 0.001$, **** $p < 0.0001$.

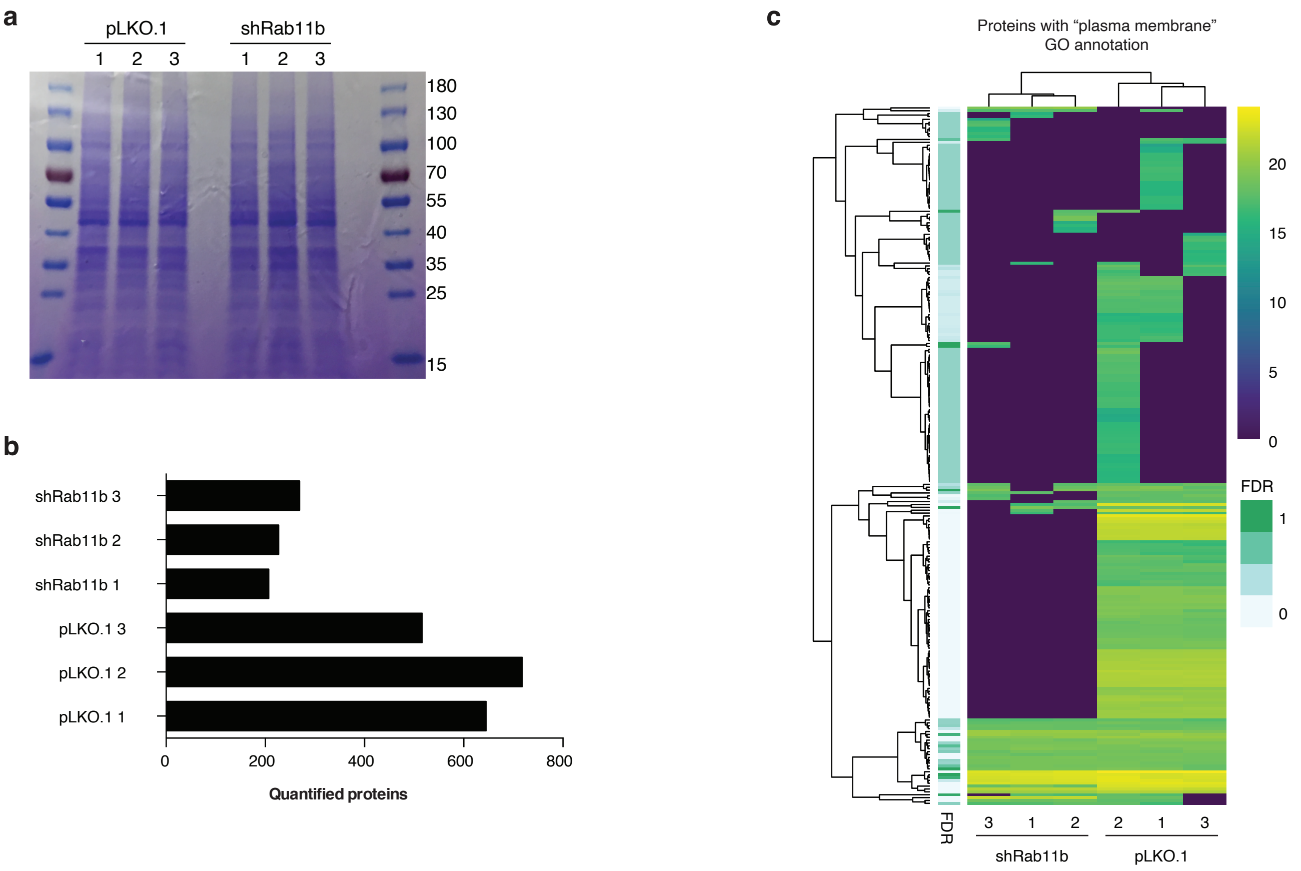

Supplementary Figure 7. Cell surface proteome analysis. Related to Figure 4.
a, Surface biotinylated MDA-231 cells were immunoprecipitated, run on a protein gel and coomassie stained.
b, Total number of proteins identified in each sample.
c, Heatmap showing all identified proteins that are annotated with the GO term plasma membrane.

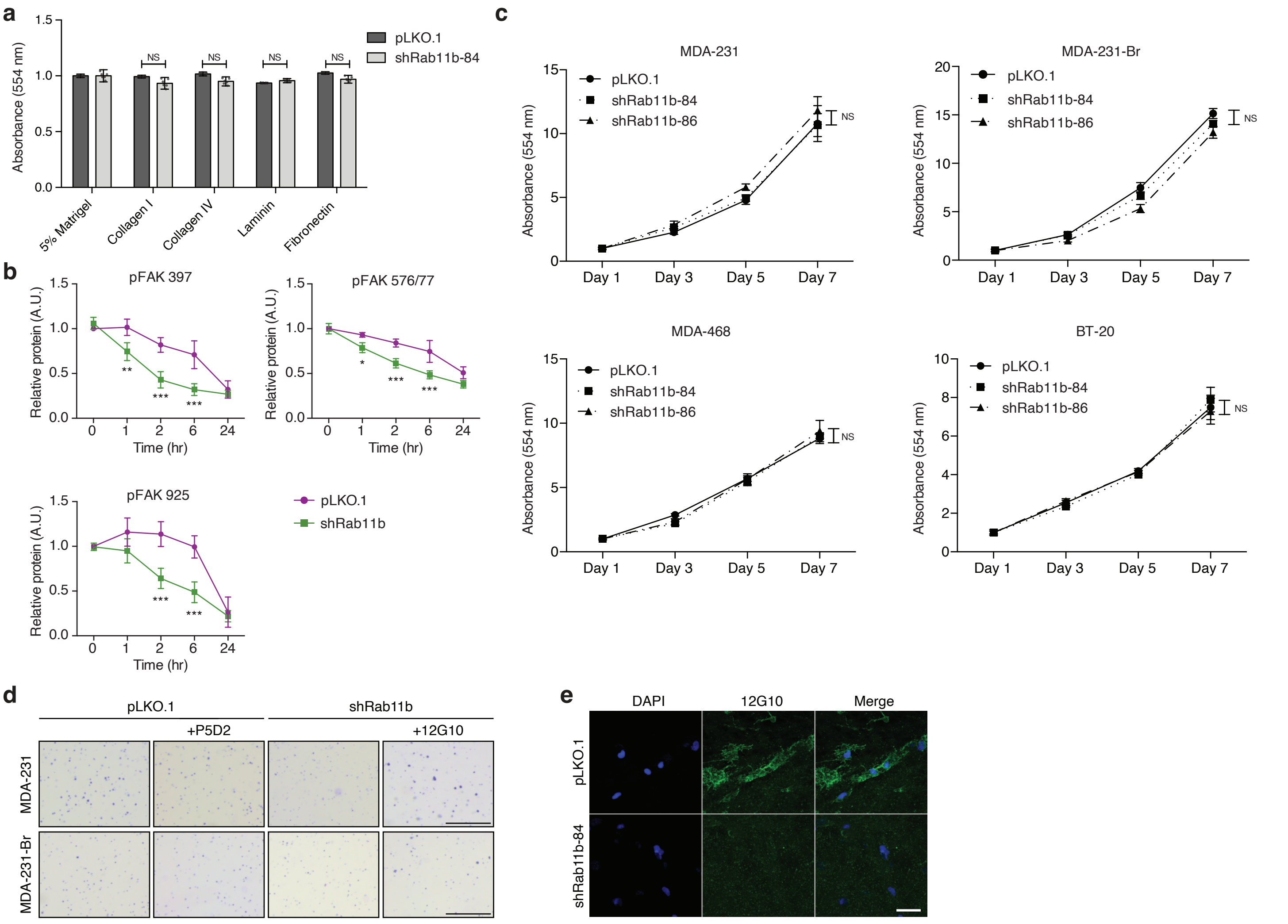

Supplementary Figure 8. Rab11b control of adhesion and growth. Related to Figure 5.

a, Adhesion assay for MDA-231 cells given 48 hrs to attach. Each cell line normalized to Matrigel control. n = 3. Mean ± s.d. Two-way ANOVA with Sidak's multiple comparison test.

b, Quantication of pFAK in MDA-231-Br immublots presented in Figure 5C, presented relative to FAK normalized to actin for three independent experiments. Mean ± s.d. Two-way ANOVA with Sidak's multiple comparison test.

c, Cell proliferation. n = 3. Mean ± s.d. Two-way ANOVA with Sidak's multiple comparison test.

d, *Left*, Representative images of cells grown for 2 weeks in soft agar in the presence of P5D2 or 12G10. Scale bar 1 mm.

e, Representative images of MDA-231 cells allowed to adhere to decellularized brain matrix for 48 hr, and stained for active integrin β1 (12G10, green), and nuclei (DAPI, blue). Scale bar 50 μm

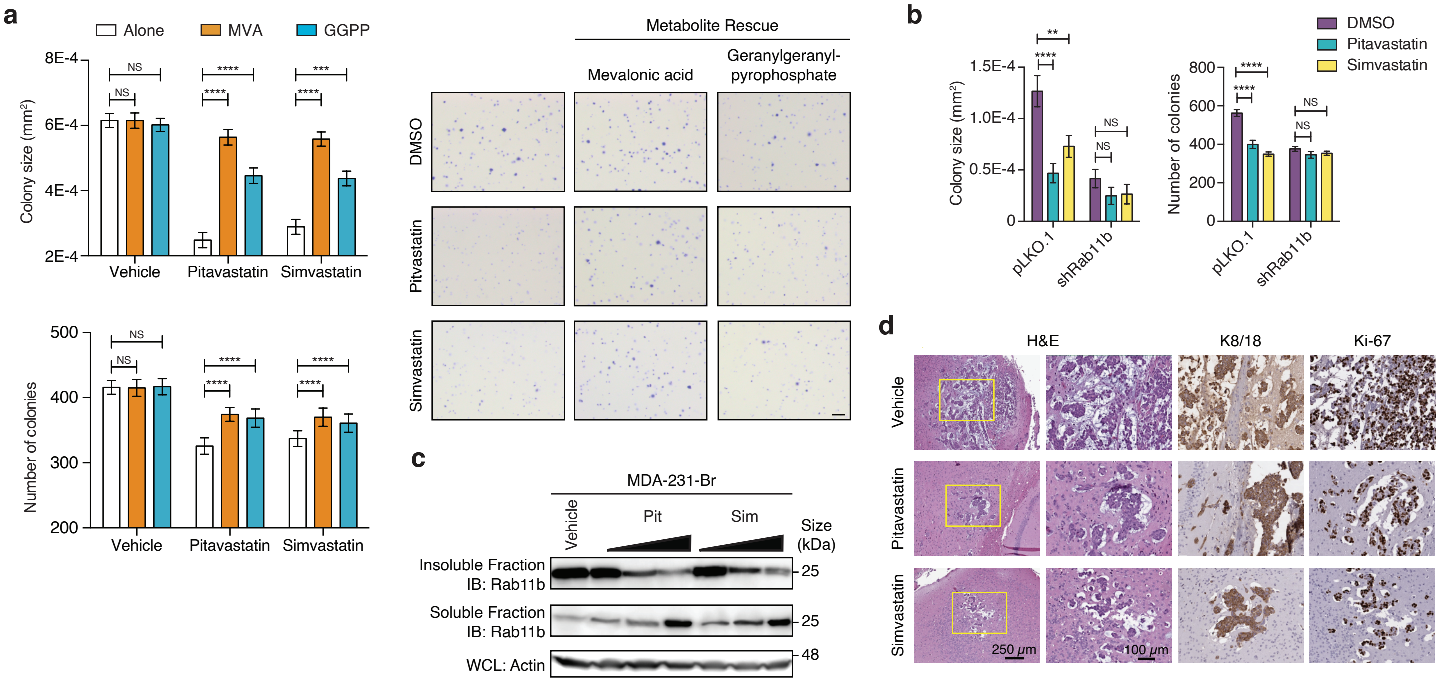

Supplementary Figure 9. Statin inhibition of Rab11b and breast cancer brain metastasis. Related to Figures 6 and 7.

a, MDA-231-Br cells were grown in soft agar with 1 µM pitavastatin or simvastatin, with the addition of 100 µM mevalonic acid or 10 µM geranylgeranylpyrophosphate for two weeks. Colonies were fixed, stained and imaged. *Left*, quantification of colony number and size. n = 10 fields per 3 independent experiments. Mean ± s.d. Two-way ANOVA with Sidak's multiple comparison test. *Right*, representative images. Scale bar 1 mm.

b, MDA-231-Br cells were grown in soft agar with 1 µM pitavastatin or simvastatin. n = 10 fields per 3 independent experiments. Mean ± s.d. Two-way ANOVA with Sidak's multiple comparison test.

c, MDA-231-Br cells grown in the presence of vehicle or 10 µM-100 nM pitavastatin or simvastatin for 24 hrs. Soluble and insoluble fractions were separated with Triton X-114, and subjected to immunoblotting.

d, MDA-231-tdTomato cells were intracranially injected, and given daily intraperitoneal injections of vehicle or 1 mg/kg pitavastatin or 5 mg/kg simvastatin. Animals were sacrificed 34 dpi. Representative images of H&E, cytokeratin 8/18 and Ki-67 immunostaining.

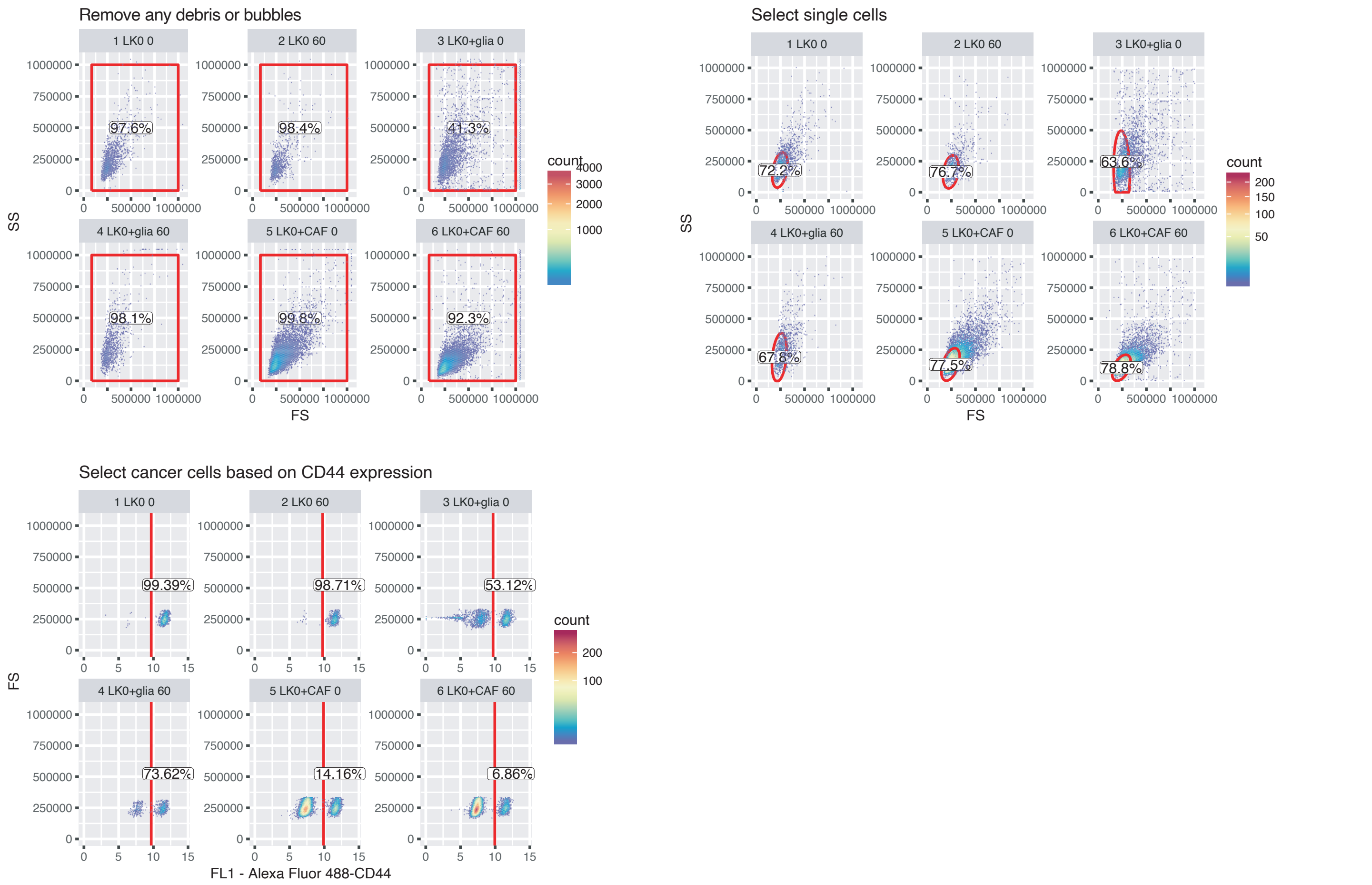

Supplementary Figure 10. Flow cytometry gating strategy.
MDA-231-pLKO.1 cells cultured alone or co-cultured with glial or CAF cells were labeled with Transferrin-Alexa-647 for internalization and recycling assay as described in methods. Samples were stained with Alexa-488-CD44 to select for MDA-231 cells. Following selection of single cells, Alexa-488-CD44 signal was used to identify MDA-231 cells as indicated. Total FL4-Alexa-647 signal was then quantified for all positively selected cells.
