# Supplementary material for: Rab11b-mediated integrin recycling promotes brain metastatic adaptation and outgrowth
Source: Nat Commun. 2020 Jun 15;11:3017. doi: 10.1038/s41467-020-16832-2 (PMC7295786; doi:10.1038/s41467-020-16832-2)
Supplement: Supplementary file 3 — Reporting Summary [file 41467_2020_16832_MOESM3_ESM.pdf]

nature research

Corresponding author(s): Siyuan Zhang
Erin N. Howe

Last updated by author(s): 05/12/202

Reporting Summary

Nature Research wishes to improve the reproducibility of the work that we publish. This form provides structure for consistency and transparency in reporting. For further information on Nature Research policies, see Authors & Referees and the Editorial Policy Checklist.

Statistics

For all statistical analyses, confirm that the following items are present in the figure legend, table legend, main text, or Methods section.

n/a	Confirmed	
☐	☒	The exact sample size (n) for each experimental group/condition, given as a discrete number and unit of measurement
☐	☒	A statement on whether measurements were taken from distinct samples or whether the same sample was measured repeatedly
☐	☒	The statistical test(s) used AND whether they are one- or two-sided<br>*Only common tests should be described solely by name; describe more complex techniques in the Methods section.*
☐	☒	A description of all covariates tested
☐	☒	A description of any assumptions or corrections, such as tests of normality and adjustment for multiple comparisons
☐	☒	A full description of the statistical parameters including central tendency (e.g. means) or other basic estimates (e.g. regression coefficient) AND variation (e.g. standard deviation) or associated estimates of uncertainty (e.g. confidence intervals)
☐	☒	For null hypothesis testing, the test statistic (e.g. F, t, r) with confidence intervals, effect sizes, degrees of freedom and P value noted<br>*Give P values as exact values whenever suitable.*
☒	☐	For Bayesian analysis, information on the choice of priors and Markov chain Monte Carlo settings
☒	☐	For hierarchical and complex designs, identification of the appropriate level for tests and full reporting of outcomes
☒	☐	Estimates of effect sizes (e.g. Cohen's d, Pearson's r), indicating how they were calculated

Our web collection on statistics for biologists contains articles on many of the points above.

Software and code

Policy information about availability of computer code

Data collection	No data collection software was used
Data analysis	For RNA seq analysis, base calling was performed using Real Time Analysis (RTA) v1.17.21.3 (Illumina) and Bcl2FastQ conversion software v1.8.4 (Illumina). Trimmed reads were aligned to reference human and mouse transcriptomes (Figure S1). Differential expression was conducted using the cufflinks (v2.1.0) and DESeq2 (v1.4.5) pipelines. Heatmaps were generated using ggplot2 (v3.3.0).<br>Drosophila orthologs were identified using the Drosophila RNAi screening center (DRSC) Integrative Ortholog Prediction Tool (DIOPT), and RNAi fly lines were identified using the DRSC Updated Targets of RNAi Reagents tool (UP-TORR).<br>Flow cytometry analysis was performed using the FlowCore package in R.<br>LC-MS/MS data were analyzed using MaxQuant software (v. 1.6.2.10) with the Andromeda search engine. Spectra were searched against the human protein sequence database from UNIPROT (https://www.uniprot.org/uniprot/) retrieved on 05/09/2018 and a common contaminant database for protein identification and relative quantification. Bioinformatic analysis was performed in R (version 3.6.0) using packages available through Bioconductor. ENSEMBL IDs, GO terms, and GO Slim terms were annotated using biomaRt.<br>Transmembrane domains were identified using the prediction algorithm TMHMM. Heatmaps and Cleveland plots were generated using ggplot2.<br>All image processing and analysis was done in ImageJ and FIJI.<br>Statistical analysis was performed using GraphPad Prism (version 6) and R (version 3.6.0).

For manuscripts utilizing custom algorithms or software that are central to the research but not yet described in published literature, software must be made available to editors/reviewers. We strongly encourage code deposition in a community repository (e.g. GitHub). See the Nature Research guidelines for submitting code & software for further information.

Data

Policy information about availability of data

All manuscripts must include a data availability statement. This statement should provide the following information, where applicable:

- Accession codes, unique identifiers, or web links for publicly available datasets
- A list of figures that have associated raw data
- A description of any restrictions on data availability

The raw RNA-sequencing data are deposited to the National Center for Biotechnology Information Gene Expression Omnibus (GEO) and are available under accession GSE134405. The raw LC-MS/MS data are deposited to MassIVE and are available for download via ftp://massive.ucsd.edu/MSV000084093.

Field-specific reporting

Please select the one below that is the best fit for your research. If you are not sure, read the appropriate sections before making your selection.

☒ Life sciences ☐ Behavioural & social sciences ☐ Ecological, evolutionary & environmental sciences

For a reference copy of the document with all sections, see nature.com/documents/nr-reporting-summary-flat.pdf

Life sciences study design

All studies must disclose on these points even when the disclosure is negative.

Sample size	Sample size for murine experiments was determined using standard power analysis. For the first experiment, estimated effect size was determined based on in vitro experiments. For all subsequent in vivo experiments, estimated effect size was determined based on the first experiment.<br><br>For in vitro experiments power analysis was not performed to determine sample size a priori. Standard biological sample sizes were used. For in vitro experiments involving pooled cell samples, a minimum of three samples were included to account for biological variance. For in vitro experiments involving single cell analysis (e.g. quantification of immunofluorescence within individual cells) a minimum of 10 random fields of view were imaged across the coverslip and all cells were quantified within each field.
Data exclusions	No data were excluded
Replication	Three biological repeats were performed for all experiments, and all results were replicated. All attempts at replication were successful.
Randomization	Randomization was not applicable to this study because only statin in vivo treatment experiments involved animals assigned to treatment groups and there were no known characteristics, no data on metastasis size or incidence, available to distribute animals into treatment groups.
Blinding	Researchers were blinded to experimental group or treatment for scoring of IHC intensity (Figure2a, Figure7f))

Reporting for specific materials, systems and methods

We require information from authors about some types of materials, experimental systems and methods used in many studies. Here, indicate whether each material, system or method listed is relevant to your study. If you are not sure if a list item applies to your research, read the appropriate section before selecting a response.

Materials & experimental systems

n/a	Involved in the study	
☐	☒	Antibodies
☐	☒	Eukaryotic cell lines
☒	☐	Palaeontology
☐	☒	Animals and other organisms
☒	☐	Human research participants
☒	☐	Clinical data

Methods

n/a	Involved in the study	
☒	☐	ChIP-seq
☐	☒	Flow cytometry
☒	☐	MRI-based neuroimaging

Antibodies

Antibodies used	Rab11 (Abcam, ab3612); Rab11b (Thermo-Fisher, PA5-31348); K8 (Abcam, ab53280, EP1628Y); Ki-67 (Cell Signaling Technology, 9027); ITGB1 (Cell Signaling Technology, 9699, D2E5); ITGB1 (Development Studies Hybridoma Bank, AIIB2); ITGB1 (Abcam, ab30394, 12G10); Alexa Fluor-488-CD44 (BioLegend 103015, IM7); Biotin-PE (BioLegend, 409003, 1D4-C5); ITGB1 (Abcam, ab24693, P5D2); FAK (Cell Signaling Technology 13009, D2R2E); pFAK (Cell Signaling Technology: Tyr397, 8556, D20B1; Tyr576/577, 3281, Tyr925, 3284); Erk1/2 (Cell Signaling Technology, 4695, 137F5); pErk1/2 (Cell Signaling Technology, 4370,

	D13.14.4E); GFAP (Cell Signaling Technology, 12389, D1F4Q); Actin (Cell Signaling Technology, 3700, 8H10D10); Anti-rabbit-HRP (Cell Signaling Technology, 7074); Anti-mouse-HRP (Cell Signaling Technology, 7076); Anti-rabbit-HRP (Thermo-Fisher, G21234)
Validation	K8, Ki-67, ITGB1, CD44, FAK, pFAK, Erk1/2, pErk1/2 and GFAP were validated on cell lines, cell line-derived xenografts or tissues with known expression of the marker. Rab11, Rab11b were validated using shRNA and siRNA mediated knockdown of targets.

Eukaryotic cell lines

Policy information about cell lines

Cell line source(s)	MDA-MB-231, MDA-MB-468, BT-20, BT549 and HCC38 were purchased from ATCC. MDA-MB-231-Br-EGFP was a generous gift from Patricia Steeg at the National Institute of Health (Bethesda, MD). Cancer-associated fibroblast (CAF) cell line was a generous gift from Dr. Zachary Schafer at the University of Notre Dame. HEK293FT cells were purchased from Thermo-Fisher.
Authentication	MDA-MB-231, MDA-MB-468, BT-20, BT549 and HCC38 were validated with STR profiling by Genetica Cell Line Testing. MDA-MB-231-Br-EGFP was validated as matching the parental MDA-MB-231 line. HEK293FT cells were not authenticated.
Mycoplasma contamination	All cell lines tested negative for mycoplasma contamination.
Commonly misidentified lines (See ICLAC register)	No commonly misidentified cell lines were used in this study.

Animals and other organisms

Policy information about studies involving animals; ARRIVE guidelines recommended for reporting animal research

Laboratory animals	NOD.Cg-Rag1tm1MomIL2rgtm1Wjl/SzJ (007799/NRG) and C57BL/6J (000664/Black 6) mouse lines were purchased from The Jackson Laboratory and bred in house, with breeders refreshed directly from The Jackson Laboratory annually. For all experiments, female mice aged 8-16 weeks were used.
Wild animals	No wild animals were used in this study.
Field-collected samples	No field-collected samples were used in this study.
Ethics oversight	All animal use was carried out in accordance with protocols approved by the Notre Dame Institutional Animal Care and Use Committee and were in compliance with the relevant ethical regulations regarding animal research.

Note that full information on the approval of the study protocol must also be provided in the manuscript.

Flow Cytometry

Plots

Confirm that:

- [x] The axis labels state the marker and fluorochrome used (e.g. CD4-FITC).
- [x] The axis scales are clearly visible. Include numbers along axes only for bottom left plot of group (a 'group' is an analysis of identical markers).
- [ ] All plots are contour plots with outliers or pseudocolor plots.
- [ ] A numerical value for number of cells or percentage (with statistics) is provided.

Methodology

Sample preparation	Samples were fixed with 4% PFA and stained as described in the methods.
Instrument	Beckman Coulter FC500
Software	Flow cytometry analysis was performed using the FlowCore package in R.
Cell population abundance	All cell populations contained at least 5,000 cells.
Gating strategy	For co-culture experiments, Alexa Fluor-488-CD44 was used to distinguish cancer cells from glial or CAF cells. For transferrin, surface biotin and surface integrin beta 1, the total signal was quantified for all cells in each sample.

- [x] Tick this box to confirm that a figure exemplifying the gating strategy is provided in the Supplementary Information.
